# Fecal microbiota transplantation mitigates vaginal atrophy in ovariectomized mice

**DOI:** 10.18632/aging.202627

**Published:** 2021-02-26

**Authors:** Jia Huang, Wanying Shan, Fuxia Li, Zizhuo Wang, Jing Cheng, Funian Lu, Ensong Guo, Rajluxmee Beejadhursing, Rourou Xiao, Chen Liu, Bin Yang, Xi Li, Yu Fu, Ling Xi, Shixuan Wang, Ding Ma, Gang Chen, Chaoyang Sun

**Affiliations:** 1 Cancer Biology Research Center, Key Laboratory of the Ministry of Education, Tongji Hospital, Tongji Medical College, Huazhong University of Science and Technology, Wuhan 430030, Hubei, People’s Republic of China; 2 Department of Gynecology and Obstetrics, Tongji Hospital, Tongji Medical College, Huazhong University of Science and Technology, Wuhan 430030, Hubei, People’s Republic of China; 3 Department of Gynecology, Zhongnan Hospital of Wuhan University, Wuhan 430071, Hubei, People’s Republic of China

**Keywords:** genitourinary syndrome of menopause, vulvovaginal atrophy, ovariectomized mouse model, vaginal epithelium, gut microbiota

## Abstract

Vulvovaginal atrophy (VVA) is a common menopause-related symptom affecting more than 50% of midlife and older women and cancer patients whose ovarian function are lost or damaged. Regardless of estrogen deficiency, whether other factors such as the gut microbiota play role in VVA have not been thoroughly investigated. To this end, we performed ovariectomy on 12-weeks’ old mice and follow-up at 4 weeks after ovariectomy, and observed atrophied vagina and an altered gut microbiota in ovariectomized mice.. We further performed fecal microbiota transplantation with feces from another cohort of ovary-intact fecund female mice to the ovariectomized ones, and found that the vaginal epithelial atrophy was significantly alleviated as well as the gut microbiota was pointedly changed. All these results suggest that ovarian activity has some influence on the gut microbiota, and the latter from the ovary-intact female mice can somehow make the vagina of mice deficient in ovarian function healthier maybe by up-expressing ESR1 in vaginal cells and enhancing regeneration in vagina. This kind of association between gut microbiota and vaginal health need further exploration such that it may provide an alternative treatment by modulating gut microbiota in patients suffering from VVA but may be reluctant to hormone therapy.

## INTRODUCTION

Genitourinary syndrome of menopause (GSM) [1], also known as vulvovaginal atrophy (VVA), is a chronic, progressive vulvovaginal, sexual, and lower urinary tract condition characterized by a host of symptoms secondary to a clinical state of hypoestrogenism after onset of menopause [2]. More than 50% of midlife and older women complain of GSM symptoms including dryness, irritation, itching, dysuria, and dyspareunia of menopause-related vuval and vaginal atrophy [3–5]. Besides, there are also many young women suffering from GSM due to iatrogenic ovarian injury. For example, breast and gynecologic cancer patients whose ovarian functions are impaired because of oophorectomy, chemotherapy, radiation, and hormonal therapy, may experience more earlier onset of GSM with more severe GSM symptoms. Estrogen deficiency is highly associated with GSM and even regarded as the cause of GSM [1]. However, postmenopausal women, whose estrogen levels are natural low, are not necessarily to be affected by GSM. What’s more, factors such as smoking [6] and non vaginal delivery are also considered as risk factors contribute to GSM. Considering the fact that not all postmenopausal women suffer from GSM and there are some other risk factors for GSM beyond menopause, it is noteworthy to explore some more potential risk factors of GSM and thus develop more measures to alleviate the symptoms.

Some researchers have explored GSM- or VVA-related factors from a unique perspective. For example, Brotman, R.M. et al have explored the association between vaginal microbiota and VVA and concluded that low relative abundance of *Lactobacillus* in the vagina was associated with VVA [7]. This study implies that a good vaginal microbiota homeostasis ultimately improve vaginal health. In good accordance with this, another research, which surveyed the vaginal communities of most postmenopausal women receiving hormone therapy, pointed out that the vaginal microbiota of patients with hormone therapy was sharply contrasting with that of patients without hormone therapy [8]. The vaginal communities of postmenopausal women receiving hormone therapy were dominated by species of lactobacilli while that of postmenopausal women without hormone therapy relatively lacked lactobacilli and had about 10-fold fewer total bacteria. These researches collectively imply that the vaginal microbiota is highly associated with vaginal healthy status, and the vaginal microbiota, an important part of human microbiota, somehow can be modulated by estrogen, which is a vital hormone mostly derived from functioning ovaries of reproductive age female.

It has been widely accepted that the gut microbiota influences many aspects of health and plays a key homeostatic role in normal function of physiologic processes including nutrition, digestion, growth, inflammation, immunity, and protection against foreign pathogens [9, 10].

The non-pathogenic commensal bacteria influence physiological and homeostatic status in the host by regulating activity of corresponding pathways, in particular the molecular mechanisms of host cell and microbe cross-talk. For example, certain taxa of enteric commensals can stimulate cellular signaling via the generation of reactive oxygen species (ROS) in the gut epithelia and induce downstream effects including cell proliferation [11, 12]. To the best of our knowledge, the association between gut microbiota and vaginal healthy status has not been explored so far. However, there are some clues indicating the possibility that gut microbiota takes part in modulating homeostasis of reproductive tract. For example, it is reported that gut microbes with ß-glucuronidase activity modulate the estrogen enterohepatic circulation [13] by promoting the conjugated estrogens to turn into deconjugated estrogens and then to be reabsorbed into the circulation [13, 14] and thus allow the estrogens exert its biological effects longer. Furthermore, some researches focusing on evaluating effectiveness of probiotics for vaginal health also indicate potential association between gut microbiota and vaginal health. Oral probiotics could reduce recurrence for females with yeast infections [15] and females with bacterial vaginosis [16].

It is therefore likely that the gut microbiota play an important role in maintaining vaginal healthy status.

In this study, we have employed an ovariectomized mouse model to explore whether iatrogenic ovarian aging has an influence on the gut microbiota and the role of fecal microbiota transplantation in the positive feedback effects on vagina.

## RESULTS

### Ovariectomy results in vaginal atrophy in mice

**Figure 1:**
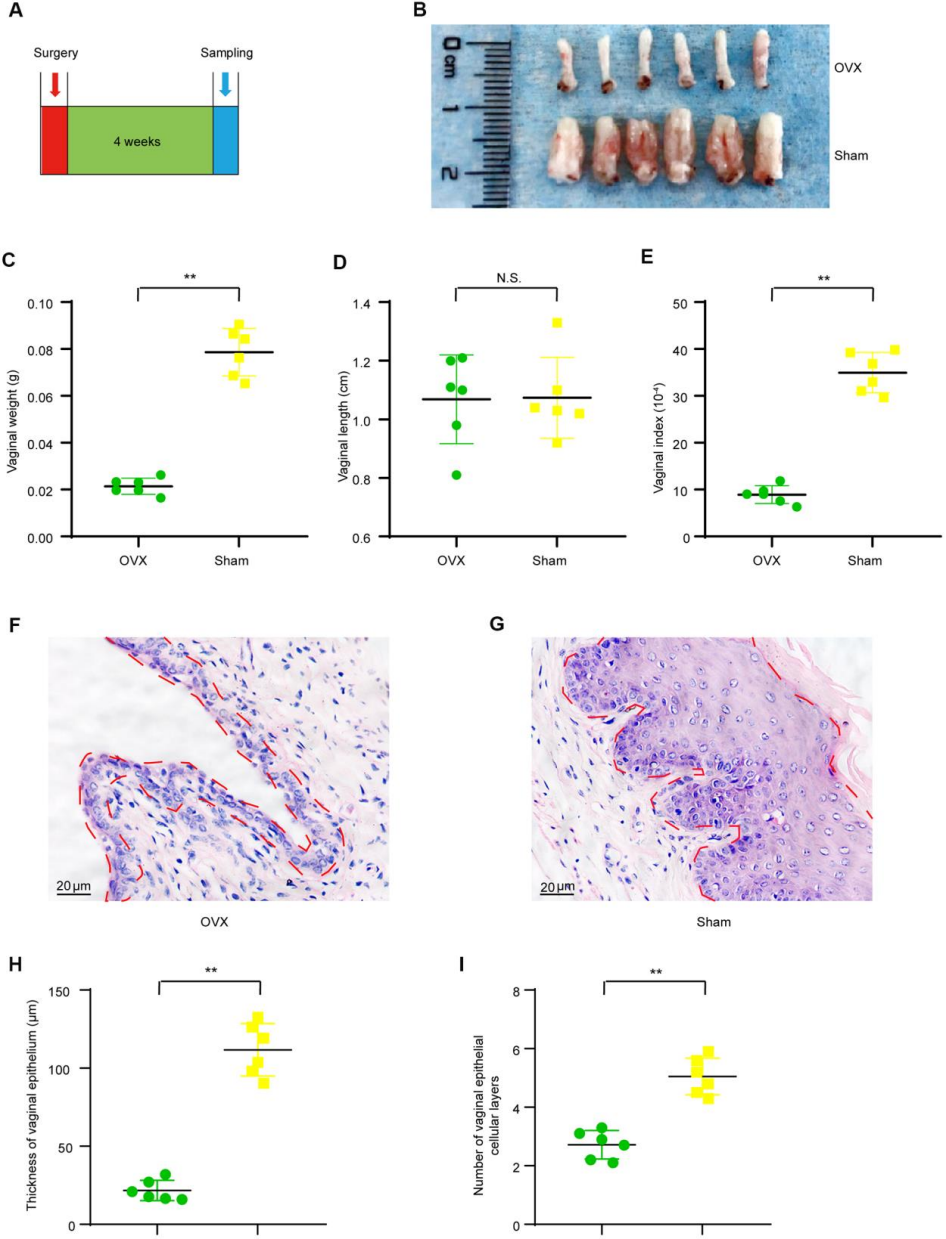
**Vaginal morphologic changes in OVX group and Sham group mice.** (**A**) The timeline for ovariectomy and sham surgery, fecal and vaginal tissue samples collection. (**B**) Representative images for isolated vagina of OVX group and Sham group. (**C**) Vaginal weight (t-test, *P*<0.05). (**D**) Vaginal length (t-test, *P*>0.05). (**E**) Vaginal index (vaginal weight/body weight*10-4) (t-test, *P*<0.05). (**F**, **G**) Representative image of Hematoxylin-Eosin staining for vaginas and the area delineated by the red dotted line indicating the epithelial layer of the vagina. (**H**) Average vaginal epithelial thickness (t-test, *P*<0.05). (**I**) Average number of vaginal epithelial cellular layers (t-test, *P*<0.05). Data are shown as mean ± SEM, n=6 for each group,*P < 0.05, **P < 0.01. OVX : bilateral ovariectomy group; Sham : sham operation group.

It has been well accepted that the ovary is the domain source of estrogen hormone in females and the estrogen hormone has various biological effects on both systemic and organ health, including the reproductive system. Therefore, ovariectomy could theoretically cause functional and morphological change in the reproductive tract. We observed that vulvas of OVX mice were atrophic and the vaginal orifices got closed naturally soon after surgery. We first evaluated if 4-weeks’ period after ovariectomy surgery is enough to induce a significant vaginal atrophy as presented in the timeline (Figure 1A). As shown by the representative image of isolated vaginas, vaginas of ovariectomy group (OVX group) were small than those of sham operated group (Sham group) 4 weeks after modeling surgery (Figure 1B). Furthermore, vaginal weights of the OVX group (0.021 ± 0.001 g) were significant lighter than those of the Sham group (0.079 ± 0.004 g) (Figure 1C) though no significant difference was observed in vaginal length between the OVX group (1.06 ± 0.06 cm) and the Sham group (1.07 ± 0.06 cm) (Figure 1D), and vagina index (vaginal weight/body weight *10^-4^) of the OVX group (8.90 ± 0.77) were significant lighter than those of the Sham group (34.94 ± 1.76) (Figure 1E). We also observed prominent morphological changes in the vaginal epithelium of OVX group when compared to that of the Sham group. The OVX group had thinner vaginal epithelium (21.70 ± 2.64μm) when compared to that of the Sham group (111.69 ± 6.86 μm) (Figure 1B–1D). The average number of vaginal epithelial cellular layer in the OVX group (2.7 ± 0.2) was smaller than that in the Sham group (5.1 ± 0.3) (Figure 1E). All these results indicated that 4 weeks after ovariectomy, the vaginal health status was dramatically altered and that the vagina was atrophic in ovariectomized mice.

### Ovariectomized mice have different gut microbial diversity compared to sham-operated mice

**Figure 2:**
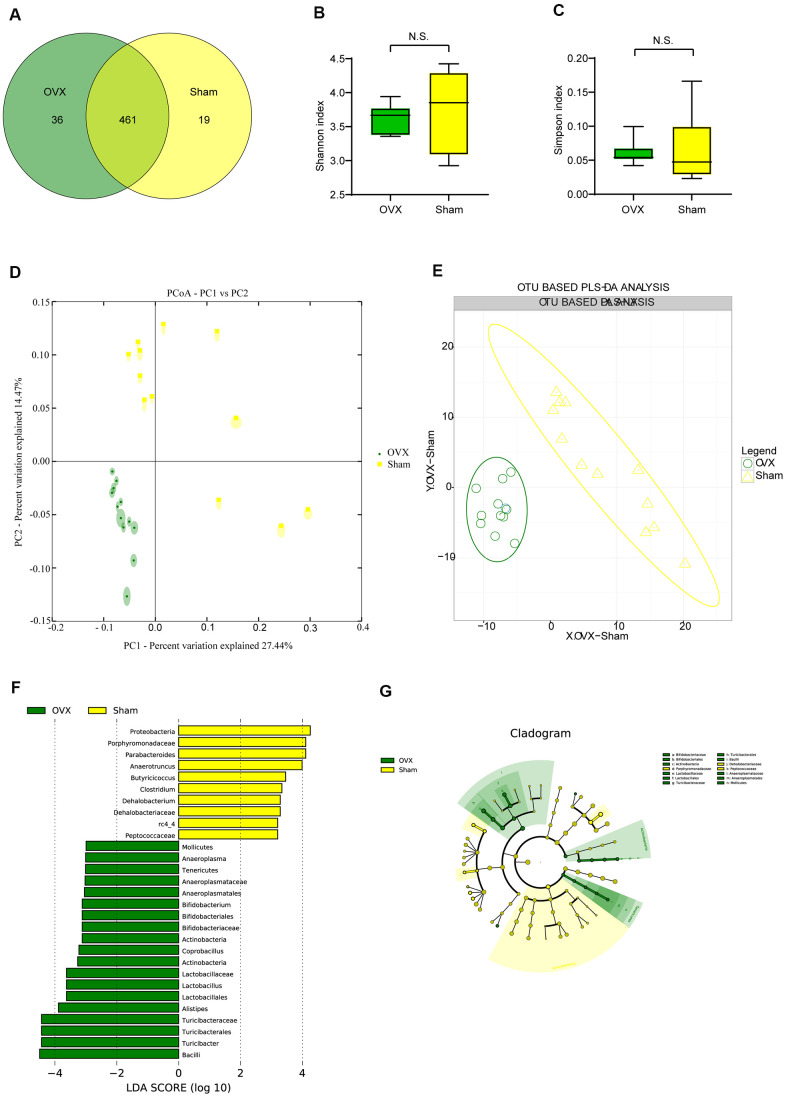
**Gut microbiota analysis in the OVX and the Sham group mice.** (**A**) Wayne diagram analysis based on OTU for the OVX group and the Sham group. (**B**) Shannon diversity index between OVX group (n=12) and Sham group (n=12) (t-test, *P*>0.05). (**C**) Simpson diversity index between OVX group (n=12) and Sham group (n=12) (t-test, *P*>0.05). (**D**, **E**) β-diversity analysis including principal component analysis (PCA) and OTU based partial least squares discrimination analysis (PLS-DA) between OVX and Sham group. (**F**, **G**) Significant differences analysis between OVX group and Sham group determined by linear discriminant analysis Effect Size (LEfSe). Data are presented as mean ± SEM. *P < 0.05, **P < 0.01. OTU: Operational Taxonomic Units; PCA: principal component analysis; PLS-DA: partial least squares discrimination analysis; *LEfSe*: Linear discriminant analysis Effect Size analysis.

We performed 16S rRNA analysis on OVX and Sham mice feces aiming at exploring whether the gut flora would change in ovariectomized female mice. Wayne diagram analysis revealed that while there were 461 Operational Taxonomic Units (OTUs) shared by the two groups, there were 36 specific OTUs in the OVX group and 19 specific OTUs in the Sham group (Figure 2A). α-diversity analysis showed that there were no significant difference of Shannon diversity index (Figure 2B) and Simpson diversity index (Figure 2C) between OVX group and Sham group while observed species (sobs) index (Supplementary Figure 1A) and Chao1 index (Supplementary Figure 1B) in OVX group were significant higher than those of the Sham group. All of these differences supported higher species diversity within the feces of OVX group and showed a tendency that ovariectomy could have an influence on the gut microbiota. OUT-based β-diversity analysis including principal component analysis (PCA) and partial least squares discrimination analysis (PLS-DA) were employed to investigate the difference of gut microbiota composition between the OVX group and the Sham group. The results of PCA (Figure 2D) and (PLS-DA (Figure 2E) illustrated a different gut microbiota between the OVX group and the Sham group.

### Ovariectomized mice have different gut microbial composition compared to sham-operated mice

**Figure 3:**
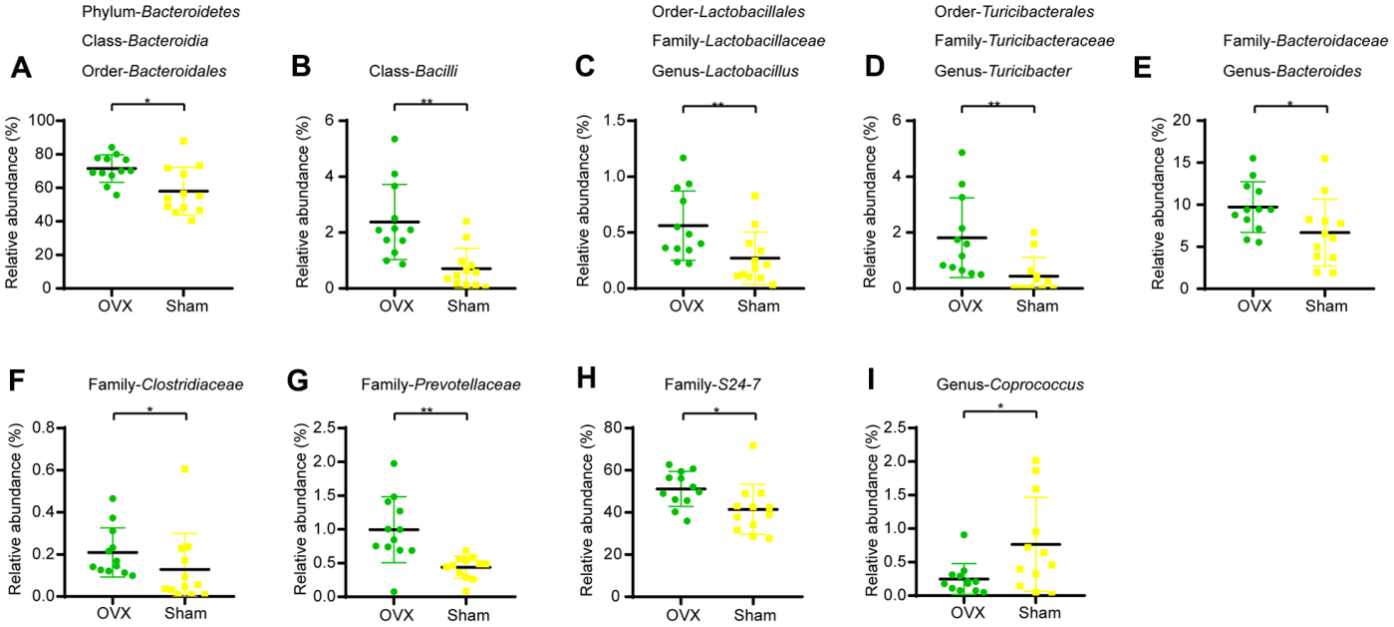
**Differential microbial categories of the gut bacteria between OVX and Sham group mice.** (**A**) Phylum-*Bacteroidetes* (t-test, *P*<0.05); Class-*Bacteroidia* (t-test, *P*<0.05); Order-Bacteroidales (t-test, P<0.05); (**B**) Class-*Bacilli* (t-test, *P*<0.01); (**C**) Order-*Lactobacillales* (t-test, *P*<0.01); Family-Lactobacillaceae (t-test, P<0.01); Genus-Lactobacillus (t-test, P<0.01); (**D**) Order-*Turicibacterales* (t-test, *P*<0.01); Family-Turicibacteraceae (t-test, P<0.01); Genus-Turicibacter (t-test, P<0.01); (**E**) Family-*Bacteroidaceae* (t-test, *P*<0.05); Genus-*Bacteroides* (t-test, *P*<0.05); (**F**) Family-*Clostridiaceae* (t-test, *P*<0.05); (**G**) Family-*Prevotellaceae* (t-test, *P*<0.01); (**H**) Family-*S24-7* (t-test, *P*<0.05); (**I**) Genus-*Coprococcus* (t-test, *P*<0.05). Data are relative abundances of certain bacterium (percentage of certain bacterium among all fecal bacteria in a mouse) and presented as mean ± SEM, n=12 for each group, *P < 0.05, **P < 0.01.

We profiled the composition of gut microbiota at the genus level for the OVX group and the Sham group (Supplementary Figure 1C) and performed clustering heat map analysis at genus level based on the Log10 value of relative abundance of each microbial categories at genus level (Supplementary Figure 1D). As shown by the bar plots of microbes at genus level (Supplementary Figure 1C), *Akkermansia, Bacteroides, Prevotella, Parabacteroides, Sutterella, Oscillospira, Turicibacter* and *Ruminococus* were the main categories in both OVX group and Sham group. And clustering heat map analysis showed separation trends between the microbial composition of OVX group and that of the Sham group. We further employed linear discriminant analysis Effect Size (LEfSe) analysis to determine the microbial categories most likely to explain differences between the OVX group and the Sham group by coupling standard tests for statistical significance with additional tests encompassing biological consistency and effect relevance (Figure 2F, 2G) and we further showed these differentially distributed microbial categories whose relative abundance were more than 0.1% (Figure 3A–3P). Briefly, there were 15 categories were more abundant in OVX group and 1 category was more abundant in Sham group (Figure 3A–3P).

### Lavage with feces from normal reproductive female mice relieve the condition of vaginal atrophy of OVX mice

**Figure 4:**
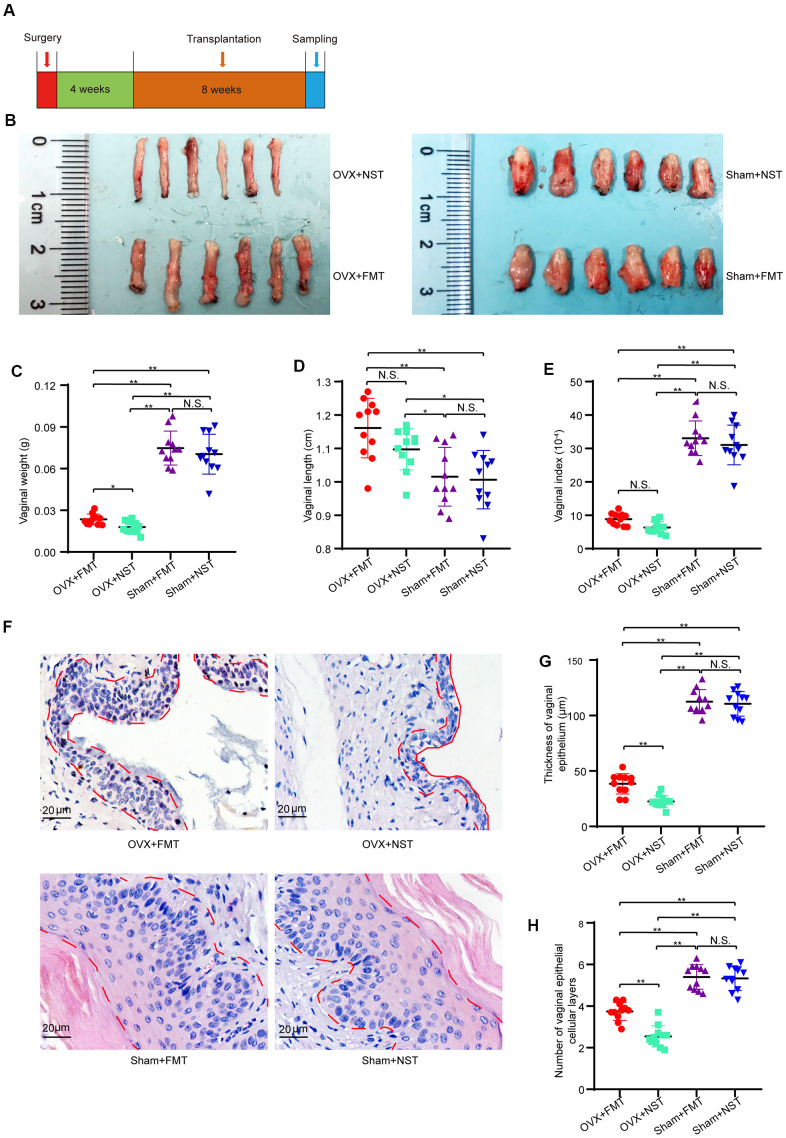
**Fecal transplantation relieves vaginal atrophy in OVX+FMT mice.** (**A**) The timeline of fecal transplantation and sample collection for OVX+NST and OVX+FT mice. (**B**) Representative images for isolated vagina of OVX+FMT, OVX+NST, Sham+FMT and Sham+NST group. (**C**) Vaginal weight (one-way ANOVA, F_3,40_=101.07, *P*<0.001), (**D**) Vaginal length (one-way ANOVA, F_3,40_=8.68, *P*<0.001). (**E**) Vaginal index (vaginal weight/body weight*10-4) (one-way ANOVA, F_3,40_=129.36, *P*<0.001). (**F**) Representative of eosin and hematoxylin staining for vagina of OVX+NST, OVX+FT, Sham+FMT and Sham+NST group mice (magnification 200x), the area delineated by the red dotted line denotes the epithelial layer of the vagina. (**G**) Average vaginal epithelial thickness of the respective OVS+NST, OVS+FT, Sham+FMT and Sham+NST groups (one-way ANOVA, F_3,40_=278.45, *P*<0.001). (**H**) Average number of vaginal epithelial cellular layers of the OVX+NST group and the OVX+FT group (one-way ANOVA, F_3,40_=74.19, *P*<0.001). Data are shown as mean ± SEM, n=11 for each group, *P < 0.05, **P < 0.01. ANOVA: analysis of variance; OVX+NST: bilateral ovariectomy group with normal saline lavage; OVX+FT: bilateral ovariectomy group with fecal suspension lavage; Sham+FMT: sham operation group with fecal suspension lavage; Sham+NST: sham operation group with normal saline lavage.

To investigate the impact of gut microbiota of ovary-intact reproductive mouse on the health of vaginal epithelium, we subsequently performed fecal microbiota transplantation to ovariectomized mice (OVX+FMT group) and Sham operated mice (Sham+FMT group) or normal sterile saline transplantation to ovariectomized mice (OVX+NST group) and Sham operated mice (Sham+NST group) and compared the differences among the OVX+FMT, OVX+NST, Sham+FMT and Sham+NST groups following the procedure as shown in Figure 4A. Representative images of isolated vagina showed that the vaginal sizes of Sham+NST group and Sham+FMT group were near the same while the vaginal sizes of OVX+NST group were smaller than those of the OVX+FMT group (Figure 4B). In details, though the vaginal weights of Sham+FMT group (0.075 ± 0.004 g) and Sham+NST group (0.071 ± 0.004 g) were larger than vaginal weights of both OVX+FMT group (0.027 ± 0.001 g) and OVX+NST group (0.018 ± 0.004 g), vaginal weights of OVX+FMT group were markedly increased when compared to those of the OVX+NST group (Figure 4C). Vaginal lengths of Sham+FMT group (1.02 ± 0.03 cm) and Sham+NST group (1.01 ± 0.03 cm) were slightly shorter than vaginal weights of both OVX+FMT group (1.16 ± 0.03 cm) and OVX+NST group (1.10 ± 0.02 cm), but no difference was observed between the vaginal length of OVX+FMT group and that of the OVX+NST group (Figure 4D). Meanwhile, vaginal index of Sham+FMT group (33.05 ± 1.56) and Sham+NST group (31.05 ± 1.79) were larger than both of these of OVX+FMT group (8.84 ± 0.55) and OVX+NST group (6.32 ± 0.53), vaginal indexes of OVX+FMT group were markedly increased when compared to those of the OVX+NST group as well (Figure 4E). We also found that 8 weeks fecal transplantation therapy significantly alleviated vaginal epithelial atrophy Though the thickness of vaginal epithelium as well as vaginal epithelial layers of OVX+FMT group were significant smaller to those of both Sham+FMT group and Sham+NST group (Figure 4F–4H), average vaginal thickness of OVX+FMT group mice (49.49 ± 1.95μm) was significantly thicker than that of the OVX+NST group mice (20.16 ± 0.46μm) (Figure 4G, 4F) In good accordance with this, average number of vaginal epithelial cellular layers in the OVX+NST group (2.55 ± 0.15) was smaller than that in the OVX+FMT group (3.95 ± 0.13) (Figure 4H). These results indicate 8-weeks transplantation with feces from ovary-intact reproductive female mice can significantly relieve vaginal epithelial atrophy of ovariectomized mice.

### OVX+FT mice have different gut microbial diversity and composition when compared with OVX+NST mice

**Figure 5:**
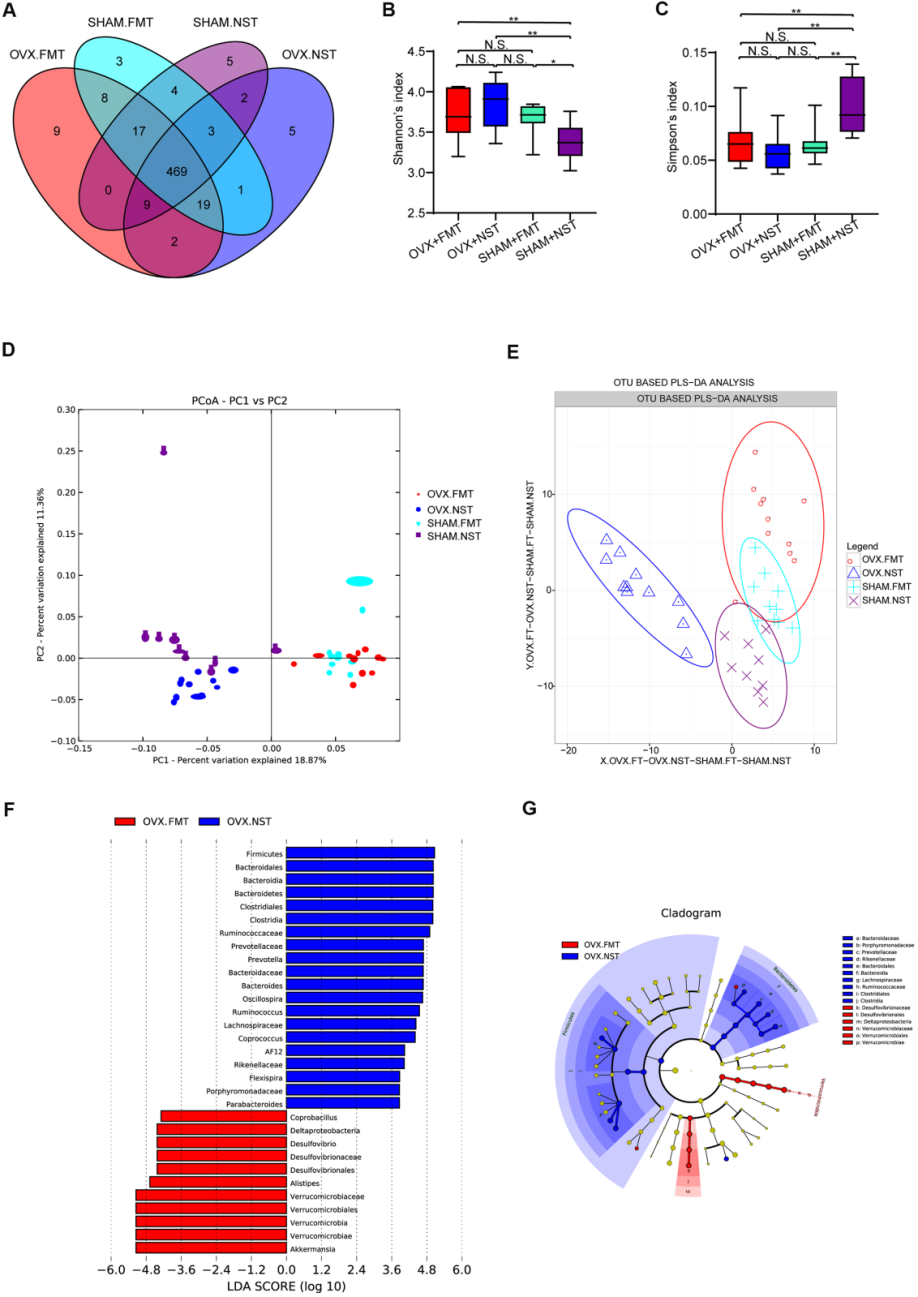
**Gut microbiota analysis for mice received fecal microbiota and normal saline lavage.** (**A**) Wayne diagram analysis based on OTU for OVX+FMT (n=11), OVX+NST (n=11), Sham+FMT (n=10) and Sham+NST (n=9) groups. (**B**) Shannon diversity index among OVX+FMT (n=11), OVX+NST (n=11), Sham+FMT (n=10) and Sham+NST (n=9) (one-way ANOVA, F_3, 37_=6.86, *P*=0.001) (**C**) Simpson diversity index among OVX+FMT (n=11), OVX+NST (n=11), Sham+FMT (n=10) and Sham+NST (n=9) groups (one-way ANOVA, F_3, 37_=8.35, *P*<0.001). (**D**, **E**) β-diversity analysis including PCA and OTU based PLS-DA for OVX+FMT (n=11), OVX+NST (n=11), Sham+FMT (n=10) and Sham+NST (n=9) groups. (**F**, **G**) Significant differences analysis among OVX+FMT (n=11), OVX+NST (n=11), Sham+FMT (n=10) and Sham+NST (n=9) groups by linear discriminant analysis Effect Size (LEfSe) analysis.

**Figure 6:**
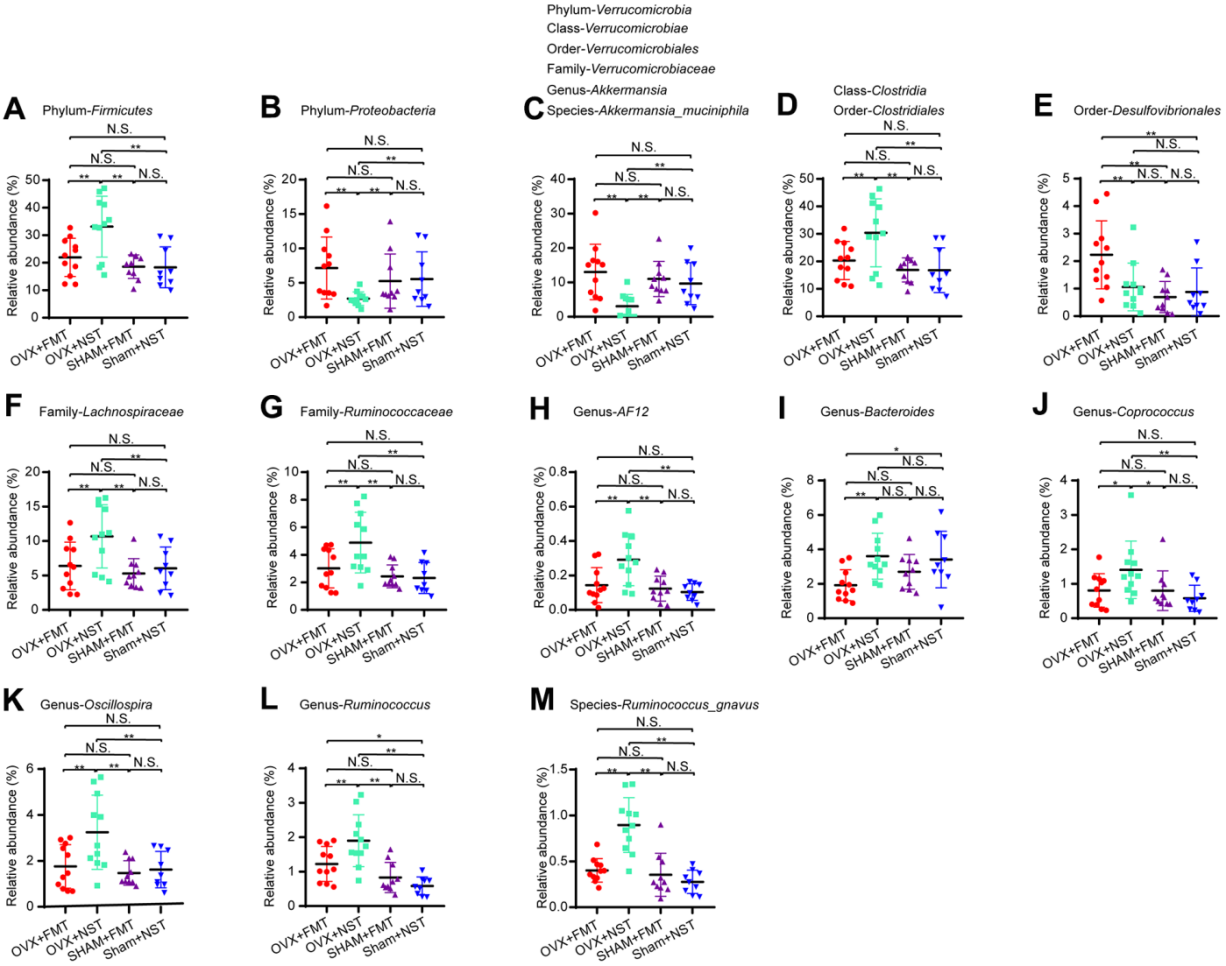
**Differential microbial categories in fecal microbiota lavage mice and normal saline lavage mice.** (**A**) Phylum-*Firmicutes* (one-way ANOVA, F_3, 37_=8.16, *P*<0.001); (**B**) Phylum-*Proteobacteria* (one-way ANOVA, F_3, 37_=8.16, *P*<0.001); (**C**) Phylum-*Verrucomicrobia* (one-way ANOVA, F_3, 37_=5.78, *P*<0.01); Class-Verrucomicrobiae (one-way ANOVA, F3, 37=5.78, P<0.01); Order-Verrucomicrobiales (one-way ANOVA, F3, 37=5.78, P<0.01); Family-Verrucomicrobiaceae (one-way ANOVA, F3, 37=5.78, P<0.01); Genus-Akkermansia (one-way ANOVA, F3, 37=5.78, P<0.01); Species-Akkermansia_muciniphila (one-way ANOVA, F3, 37=5.78, P<0.01); (**D**) Class-*Clostridia* (one-way ANOVA, F_3, 37_=5.88, *P*<0.01); Order-Clostridiales (one-way ANOVA, F3, 37=5.88, P<0.01); (**E**) Order-*Desulfovibrionales* (one-way ANOVA, F_3, 37_=5.92, *P*<0.01); (**F**) Family-*Lachnospiraceae* (one-way ANOVA, F_3, 37_=5.18, *P*<0.01); (**G**) Family-*Ruminococcaceae* (one-way ANOVA, F_3, 37_=6.42, *P*=0.001); (**H**) Genus-*AF12* (one-way ANOVA, F_3, 37_=7.17, *P*=0.001); (**I**) Genus-*Bacteroides* (one-way ANOVA, F_3, 37_=4.09, *P*<0.05); (**J**) Genus-*Coprococcus* (one-way ANOVA, F_3, 37_=3.59, *P*<0.05); (**K**) Genus-*Oscillospira* (one-way ANOVA, F_3, 37_=6.24, *P*<0.01); (**L**) Genus-*Ruminococcus* (one-way ANOVA, F_3, 37_=11.86, *P*<0.001); (**M**) Species-*Akkermansia_muciniphila* (one-way ANOVA, F_3, 37_=5.78, *P*<0.01). Data are relative abundances of certain bacterium (percentage of certain bacterium among all fecal bacteria in a mouse) and presented as mean ± SEM, n=11 for OVX+FMT group and OVX+NST group respectively, n=10 for Sham+FMT group and n=9 for Sham+NST group, *P < 0.05, **P < 0.01.

16 rDNA sequencing and analysis for fecal microbiota from OVX+FMT, OVX+NST, Sham+FMT and Sham+NST groups showed that there were 533 OTUs in OVX+FMT group, 510 OTUs in OVX+NST group, 524 OTUs in Sham+FMT group and 509 OTUs in Sham+NST group (Figure 5A). When pairwise compared to find out the shared OTUs numbers among these four groups, we found that the shared OTUs number between the OVX+FMT group and the Sham+ FMT was bigger than any others pairwise groups. When further conducted α-diversity analysis with one-way ANOVA followed by post hoc test, no significant difference was observed between OVX+FMT and OVX+NST groups for Shannon index (Figure 5B) Simpson index (Figure 5C), sobs index (Supplementary Figure 2A) and Chao1 index (Supplementary Figure 2B). However, β-diversity analysis including PCA (Figure 5D) and PLS-DA (Figure 5E) indicated that gut microbiota of the OVX+FMT group was similar to that of the Sham+FMT group but was different to that of the OVX+NST group. The PCA diagram also indicated that fecal microbiota transplantation could modulate the gut microbiota since the samples from the normal sterile saline gavage groups (including OVX+NST and Sham+NST groups) were relatively close while the samples from feces transplanted groups (including OVX+FMT and Sham+FMT groups) were close, and samples from the normal sterile saline gavage groups were relative far from samples from feces transplanted groups (Figure 5D). We further performed microbial taxonomy in OVX+FMT, OVX+NST, Sham+FMT and Sham+NST groups. The bar plots for fecal microbes at genus level for four groups showed that *Akkermansia*, *Prevotella, Bacteroides, Helicobacter, Oscillospira, Sutterella, Desulfovibrio, Ruminococus, Coprococcus and Lactobacillus* were the main categories at genus level (Supplementary Figure 2C). And samples from OVX+NST group were different from other samples as shown by the clustering heat map analysis (Supplementary Figure 2D). We further performed the LEfSe analysis to select the microbes that are distinguished to the OVX+FMT group and the OVX+NST group (Figure 5F, 5G) and displayed the differentially distributed categories whose relative abundance were above 0.1% (Figure 6A–6S). Briefly, there were 7 categories at different taxonomic rankings were more abundant in OVX+FMT group than in OVX+NST group and there were 11 categories at different taxonomic rankings were more abundant in OVX+NST group than in OVX+FMT group. Interestingly, all these differentially distributed 18 categories shared similar abundance among OVX+FMT group, Sham+FMT group and Sham+NST group. There was also only one category (Figure 6G, Order-*Desulfovibrionales*) more abundant in the OVX+FMT than the others 3 groups.

### Fecal microbiota transplantation enhances vaginal cell proliferation

**Figure 7:**
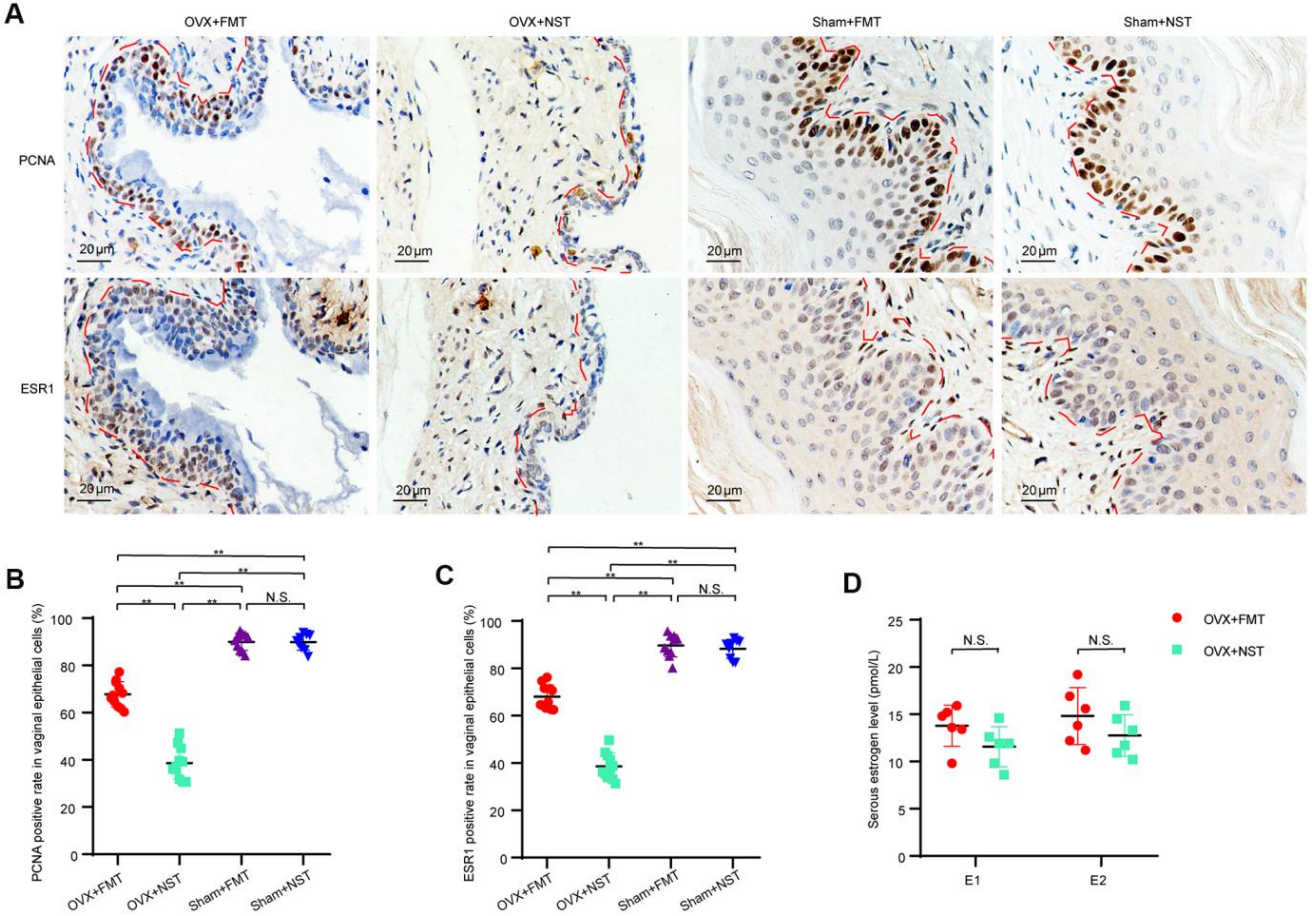
**Fecal microbiota transplantation enhances proliferation of vaginal cells.** (**A**). Representative images of PCNA and ESR1 immunohistochemical staining in vaginas from OVX+FMT, OVX+NST, Sham+FMT and Sham+ NST groups, (magnification 200x). (**B**). Positive rate of PCNA in vaginal epithelial cells in the OVX+FMT (n=11), OVX+NST (n=11), Sham+FMT (n=10) and Sham+NST (n=9) groups (one-way ANOVA, F_3, 37_=234.68, *P*<0.01). (**C**) Positive rate of ESR1 in vaginal epithelial cells in the OVX+FMT (n=11), OVX+NST (n=11), Sham+FMT (n=10) and Sham+NST (n=9) groups (one-way ANOVA, F_3, 37_=243.5, *P*<0.01). (**D**) Serum E1 and E2 level of the OVX+NST group (n=6) and the OVX+FT (n=6) group mice (t-test, *P*>0.05). Data are shown as mean ± SEM. *P < 0.05, **P < 0.01. PCNA: proliferating cell nuclear antigen; ESR1: estrogen receptor alpha; E1: Estrone; E2: Estradiol.

Since as shown in our study, vaginal epithelium is a good marker for vaginal atrophy and it is assumed that the epithelial thickness is secondary to epithelial cells proliferation, we were curious whether the vaginal epithelial proliferative capacity would be enhanced by fecal transplantation in the OVX+FMT group. We performed immunohistochemical analysis for PCNA and ESR1 (Figure 7A) on the mice vaginal sections and found that the percentage of PCNA(+) epithelial cells in the OVX+FMT group (67.8 ± 1.6) was visibly higher than that in the OVX+NST group (38.6 ± 2.0), and the percentage of PCNA(+) epithelial cells in both of these two groups were lower than those of Sham+FMT(89.9 ± 1.2) and Sham+NST group (89.8 ± 1.1)(Figure 7B), which indicated that fecal transplantation could somehow enhance epithelial proliferative capacity in ovariectomized mice. Similarly, the percentage of ESR1(+) epithelial cells in the OVX+FMT group (68.0 ± 1.5) were significant higher than that of the OVX+NST group (38.1 ± 1.7), and the percentage of ESR1(+) epithelial cells in Sham+FMT(89.6 ± 1.5) and Sham+NST group (88.2 ± 1.3) were near the same (Figure 7B). We further analyzed serum estrogen believing that estrogen can powerfully promote cellular proliferation in the reproductive tract. But the levels of serum estrogen including estrone (E1) and estradiol (E2) in the OVX+FMT group was not significantly different from those of the OVX+NST group (Figure 7D) (t-test, P>0.05).

## DISCUSSION

This study indicates that the removal of the ovaries in adult female mice is associated with vaginal atrophy including decreased weight of vagina, decreased vaginal index, thinned the thickness of vaginal epithelium, lessened layers of vaginal epithelium as well as altered gut microbiota. Transplanting feces from ovary-intact reproductive female mice can ameliorate the vaginal atrophic status to some degree accompanied by an alteration in gut microbiota. It is likely to conclude that normal ovaries contribute in modulating the gut microbiota in mice; and the gut microbiota in ovary-intact reproductive female mice also has a positive feedback on the vaginal epithelium.

GSM, or VVA, is a syndrome that affects more than half of postmenopausal women and many women with certain medication as well [17]. Taking survivors of breast cancer as examples, many young patients suffer from GSM due to ovarian function insufficiency or deficiency after chemotherapy, removal of ovaries or radiation. [18]. In this study, we established OVX mice to mimic patients received oophorectomy and to explore the vaginal morphologic alteration as well as the potential role of gut microbiota on vaginal health. The ovariectomized mouse model is widely used to explore menopause-related systemic situation including bone loss and osteoporosis [19, 20], hair loss [21], failure of recognition and memory [22] and so on. It is also an ideal model for researching the pathophysiologic changes in reproductive system at post menopause including female endocrine [23], uterotrophic assay [24] as well as for surveying the influencing factors for vaginal epithelial cell homeostasis [25], proliferation [26] and vaginal lubrication [27]. As far as we know, studies focusing on the issue that whether the gut microbiota plays roles in VVA are scarce. In the present study, we used the ovariectomized mouse as vagina-trophic model to explore the association of gut microbiota and iatrogenic ovarian injury-related reproductive system health. In accordance with former researches [26, 27], we have observed a significant vaginal atrophy in the OVX group since vaginal weights, lengths and vaginal indexes were lower than these in the Sham group. We have also observed a decrease in vaginal epithelial thickness and vaginal epithelial cell layers in the OVX group. These results demonstrate atrophy in the vagina, and thus confirmed that it is suitable to employ the ovariectomized mice model to investigate the healthy condition of mouse vagina.

At the same time, we have found that the gut microbiota can be largely influenced by the ovary and its function since ovariectomized mice had different gut microbiota when compared with that of the Sham mice. The altered gut microbiota may be owing to the loss of ovary in the OVX group since the only variance between the OVX group and the Sham group is the presence of the ovaries or not. Therefore, the gut microbiota of the female mouse may be moderated by the ovary somehow.

Nowadays, the fact that the genome of the microbiota inhabiting our body has been termed as the second genome of human beings [28] and the microbiome is considered as an super organ [29], has demonstrated and emphasized the importance of the interaction between hosts and microbiota. Gut microbiota, the most investigated microecology in human, has been even regarded as a virtual endocrine organ [30] or considered to be a vital regulator of human endocrine system [31]. When it comes to the topic concerning cross-talk between the gut microbiota and reproductive endocrine system, a good example can come from type 1 diabetes mellitus (T1DM). T1DM is an autoimmune disease and preferably affects females with double risks than men. Researchers have reported that testosterone acts as a protective factor for male mice and also found that sex-specific microbiome profiles emerge only after puberty [32]. These finding imply that the increasing level of testosterone after puberty begin to orchestrate alterations in the gut microbiota. Furthermore, transferring of gut microbiota from adult males to immature females altered the recipient’s microbiota by elevating testosterone and metabolomic changes, reducing islet inflammation and autoantibody production and strengthening T1DM protection [32]. Based on the fact that sex hormone can alter gut microbiota and the altered microbiota exert impact on hosts in a self-reinforcing program of sex differentiation, a new concept “Microgenderome” has been put forward to emphasize the cross-talk between the microbiota and reproductive system [33]. In accordance with these notions, the altered gut microbiota in OVX mice indicates that there is relationship between ovary and gut microbiota. Possibly, ovarian endocrine functions, for example, secretion estrogen and progesterone, could modulate gut microbiota because host’s factors can be largely modulated by these endocrine functions. The exact effects and mechanisms for ovary to modulate microbiota is worthy of further exploration.

The later fecal microbiota transplantation experiment demonstrates that gut microbiota could also take parts in maintaining the health of reproductive tract. 8-weeks course of fecal transplantation has made outcome measurements such as vaginal weights, vaginal index as well as average vaginal epithelial thickness and vaginal epithelial layers of OVX+FMT group significantly better than those of the OVX+NST group. These phenomena collectively underline that the reproductive system has been strongly influenced by fecal microbiota transplantation. In another words, the gut microbiota in fecund female mice may have some potential effects in keeping the vaginal epithelium healthy. These results, along with the finding contrasting the OVX group and the Sham group, collectively indicates that the ovarian activity of the female reproductive system has certain influence on the gut microbiota, and gut microbiota from the ovary-intact reproductive mice can provide positive feedback alone or together with other factors which assist in keeping the vagina healthy. However, when perform fecal microbiota transplantation to Sham operated mice, no significant difference of vagina-related outcome measurements is observed and no differentially distributed microbes can be found between Sham+FMT group and Sham+NST group. The similar results from Sham+FMT group and Sham+NST group further reveal that gut microbiota from ovary-intact mice are relatively steady, and also indicates that the lavage itself or lavage with normal sterile saline is less likely to induce the vaginal morphologic relief in OVX mice by secondary effect rather than by altering gut microbiota.

It is very likely fecal microbiota transplantation alleviates vaginal atrophy by enhancing cell proliferation along vagina. In accordance with our assumptions, the positive rate of PCNA in vaginal epithelial cells of the OVX+FMT group was markedly higher than that of the OVX+NST group. This finding demonstrated that the gut microbiota from ovary-intact reproductive female mouse can enhance the proliferative capacity of vaginal epithelium when transplanted into the gut of the OVX mice. Though the serous estrogen level, which is important for vaginal homeostasis is not significantly different between OVX+FMT and OVX+NST groups, the expression of ESR1 along the vagina is significant up-regulated in OVX+FMT group mouse. Considering that ESR1 is the main receptor of estrogen in vaginal epithelial [26] and is also regarded as a potential marker for vaginal epithelial progenitor cells [34], the up-regulated expression of ESR1 maybe one important mechanism for the enhanced proliferation and regeneration of vaginal epithelial cells. We thus concluded that fecal microbiota from ovary-intact mice could help to maintain vaginal homeostasis by enhancing ESR1 expression and thus enhancing the proliferative potential of vagina.

As we all know, fecal microbiota transplantation is an innovative investigational treatment for many diseases and conditions. In the current study, we performed fecal microbiota transplantation for OVX mice and alleviate the VVA signs of OVX mice at certain degree. Briefly, there are 18 microbial categories at different taxonomic rankings whose relative abundance were almost the same in OVX+FMT group, Sham+FMT and Sham+NST group. Of them, 7 microbial categories (Phylum-*Proteobacteria*, Phylum-*Verrucomicrobia*, Order-*Verrucomicrobiales*, Class-*Verrucomicrobiae*, Family-*Verrucomicrobiaceae*, Genus-*Akkermansia*, Species-*Akkermansia_muciniphila*) were more abundant in OVX+FMT group while 11 microbial categories (Phylum-*Firmicutes*, Class-*Clostridia*, Order-*Clostridiales*, Family-*Lachnospiraceae*, Family-*Ruminococcaceae*, Genus-*AF12*, Genus-*Bacteroides*, Genus-*Coprococcus*, Genus-*Oscillospira*, Genus-*Ruminococcus*, Species-*Ruminococcus_gnavus*) were less abundant in OVX+FMT group. It is likely that the 7 categories enriched in OVX+FMT group play positive role in vaginal regeneration while the 11 categories enriched in OVX+NST group play negative role in vaginal health, which need further study to confirm. Interestingly, of the 7 categories, *Akkermansia_muciniphila* as well as its 5 upper taxonomic rankings were all present and *Akkermansia_muciniphila* is regarded as an important probiotic [35]. Of the 11 categories enriched in OVX+NST group, some lower taxonomic rankings of Class-*Clostridia* are pathogenic to both humans and animals [29, 30]. However, exact roles of certain microbes should be carefully studied with some more complex experiments such as single gut microbiota transplantation to germ free animal to obtain convincing conclusions.

In summary, in this present study we have employed ovariectomized mouse model to investigate the association between gut microbiota and healthy vaginal status. We have found that ovariectomy can result in vaginal atrophy and altered gut microbiota, and when feces from ovary-intact female mice were transplanted to the ovariectomized mice, the atrophic vaginal status was largely alleviated. As indicated by the findings of the present study, we suppose that the ovarian activity has some influence on the gut microbiota and the later receiving modulation from the ovary can up-expression of ESR1 and PCNA thus act as positive feedback to make the vagina more proliferative, more regenerating and thus healthier. However, the present study has some limitation including the mechanism of how ovary modulates gut microbiota is not clear, and 16S rRNA sequencing is not sufficient for further microbiota-related mechanism exploration. Further study is needed to find out key microbes and the main mechanism for the association between ovarian activity and gut microbiota. This will probably help us in mitigating aging-related VVA by modulating the gut microbiota once safety is confirmed and guaranteed.

## MATERIALS AND METHODS

### Animal ovariectomy and sham surgeries

Young female SPF condition C57BL/6 mice (8 weeks old) were bought from Charles River Labs (Beijing, China) and housed under standard animal care conditions with free access to food and water. Experiments and animal care were performed in accordance with the guidelines and procedures authorized by the Animal Experimental Ethical Committee of Tongji Hospital, Tongji Medical College, Huazhong University of Science and Technology. After 2 weeks' adaptive feeding, mice were randomly allocated to sham-operation group (Sham group) and bilateral ovariectomy group (OVX group) and operation was performed accordingly. In brief, the mice were completely anesthetized with pentobarbital and incisions were made in the back muscle and the fat pad located just beneath the muscles was pulled out to expose the ovary. And then, the fallopian tube was clamped off, the bilateral ovary removed and muscle and skin were sutured for the OVX group (n=18) while the Sham group (n=18) underwent identical surgical procedure without the fallopian tubes being clamped off and ovaries removed. No antibiotic was added to food or drink during the surgical and wound healing processes. Mice would be screened out if there was any evidence that showed wound infection or wound bad healing to avoid microbiota disturbance.

### Fecal and vaginal tissue samples collection

4 weeks after surgery, the mice were placed in a clean cage covered with sterile filter paper and the newly excreted feces were collected as previously described [36] with sterile tubes for both Sham and OVX groups and frozen in -80° C refrigerator immediately for further microbiota analysis. Additionally, six mice from each group were randomly chosen to be euthanized for vaginal dissection. Body weights, isolated vaginal weights and length were measure by electronic scale and ruler. The upper 1/3 of the vagina was fixed in formalin, dehydrated and paraffin-embedded for subsequent histological analysis.

### Fecal transplantation for OVX mice

To further explore whether and how the ovary status can influence the gut flora and potential effects of the gut flora on the vaginal health, we performed transplantation experiments on ovariected and sham operated mice. Briefly, another cohort of young mice whose ovaries were not meddled with (n=15) were recruited as fresh feces’ donors. The fresh feces were collected and suspended with normal sterile saline with fixed concentration at 1-gram feces per milliliter to make a suspension, then the suspension was filtered by a fertile 30 mesh sieve before lavage. OVX group mice (hereinafter named OVX+FT group, n=12) and Sham group mice (hereinafter named Sham+FT group, n=10) were randomly selected to receive lavage with 200ul fecal suspension, and another OVX group mice (hereinafter named OVX+NST group, n=12) and Sham group mice (hereinafter named Sham+NST group, n=10) were treated with 200ul normal sterile saline lavage as control. The transplantation experiments were performed every 3 days and stopped after 8 weeks since we noticed that the closed vaginal orifices after ovariectomy were open again in about half of OVX+FMT group mice. 3 days after the last lavage cycle, mice were starved for 12 hours before fecal, plasmatic and vaginal tissue samples were collected for all those mice. Body weights, vaginal weights and lengths are measured as described, and upper 1/3 of the vagina was fixed, dehydrated and paraffin-embedded for subsequent histological analysis. Feces, plasma were frozen in -80° C refrigerator immediately for further test.

### Histological and immunohistochemical analysis

Paraffin-embedded transverse sections (5 μm) from formalin-fixed vaginal specimens were stained with Hematoxylin-Eosin staining to evaluate the thickness of the vaginal mucosa of the OVX, Sham, OVX+NST, OVX+FT, Sham+NST and Sham+FT groups. The thickness of the vaginal mucosa were measured with the help of software Image J in two transverse vaginal sections for each specimen, and each section had 10 measurements from the basal membrane to the apical surface under corresponding image acquired by BX53F microscope (Olympus Corporation, Tokyo, Japan) (200 x) as described in a previous report [26] and the mean of these measurements represents the average thickness of vaginal mucosa as described by Benoit [37]. Number of vaginal epithelial cellular layers was counted as detailed by Hu Xiang [38].

Additionally, antibody for PCNA and ESR1 was employed for immunohistochemical staining to detect the expression of PCNA and ESR1 in vaginal mucosal cell. After de-paraffinization, rehydration, and antigen retrieval, the sections were incubated with anti-PCNA(1:200, Abclonal, catalog: A0264, China) antibody and anti-ESR1(1:200, Abclonal, catalog: A0296, China) overnight at 4° C, followed by detection using the DAB system (ZSGB-BIO, Beijing, China) and staining with haematoxylin. Images were acquired for each section using a BX53F microscope (Olympus Corporation, Tokyo, Japan). Average numbers of PCNA-positive as well as ESR1-positive vaginal mucosal cells and the total epithelial cells of the respective field were counted with the help of Image J software, and 5 fields of a vaginal section under a microscope (400 x) were recorded, and the average percentage of PCNA (+) or ESR1(+)of vaginal epithelial cells was calculated by dividing the average number of PCNA-positive or ESR1-positive vaginal epithelial cell by the total vaginal epithelial cells number for each vaginal section.

### Serum Estrogen detection by ELISA

We performed ELISA to detect estrone (E1) and estradiol (E2) following the guidance of the manufacturer of ELISA kits (Elabscience, Wuhan, China). Briefly, 50 uL of serum samples from the OVX group and the Sham group and a reference standard provided by the kits were added to micro ELISA plates and assay was carried out and the results calculated according to the product manual. The enzyme-substrate reaction was terminated by the addition of stop solution and the color change was measured spectrophotometrically at a wavelength of 450 nm ± 2 nm. The concentration of E1 and E2 in the samples was then determined by comparing the OD of the samples to the standard curve.

### 16S rRNA analysis of mice feces

The 16S rRNA analysis of the mice feces was performed and analyzed by BGI Co., Ltd., (Shenzhen, China). In summary, bacterial genomic DNA was extracted with CTBA and quality tested, and only qualified DNA would be amplified in 50 μL triplicate reactions with bacterial 16S rRNA gene (V4 region)-specific primers: 515F (5′- GTGCCAGCMGCCGCGGTAA -3′) and 806R (5′-GG ACTACHVGGGTWTCTAAT-3′). Then the jagged ends of PCR product DNA were converted into blunt ends by using T4 DNA polymerase, Klenow Fragment and T4 Polynucleotide Kinase. Next, an 'A' base was added to each 3' end to make it easier for addition of adapters. Afterwards, fragments too short were removed by Ampure beads. For genomics DNA, fusion primer with dual index and adapters was used for PCR and fragments too short were also discarded by Ampure beads. In both cases, only the qualified library would be used for sequencing on an Illumina Miseq PE250 system. Briefly, libraries of 12 samples for OVX group, 12 samples for Sham group, 11 samples for OVX+FMT group, 11 samples for OVX+NST group, 10 samples for Sham+FMT group and 9 samples for Sham+NST group were qualified and further sequenced. The original sequencing reads were filtered to obtain clean reads, and then paired-end reads with overlap were merged into tags. And tags were clustered to OTU by scripts of software USEARCH (v7.0.1090) [39] at a 97% threshold by using UPARSE. Taxonomic ranks were assigned to OTU representative sequence by using Ribosomal Database Project (RDP) Classifier v.2.2 [40] trained on the Greengenes database. Lastly, α-diversity calculated by Mothur (version: 1.31.2), β-diversity calculated by QIIME pipeline [41] and the different species screened by LEfSe software [42] were all performed based on OTU and taxonomic ranks.

### Statistical analysis

All quantitative *data* are expressed as means (M) ± standard error of the mean (SEM). Student’s *t*-test was used to compare the difference of means between two groups, and one-way analysis of variance (ANOVA) followed by post hoc Tukey’s test was used to compare means among groups. All these analyses were performed by using SPSS software version 19.0 (SPSS Inc., Armonk, New York, USA). *P*-values lower than 0.05 were considered statistically significant.

## Supplementary Material

Supplementary Figures
