# Supplementary material for: Fecal microbiota transplantation mitigates vaginal atrophy in ovariectomized mice
Source: Aging (Albany NY). 2021 Feb 26;13(5):7589–607. doi: 10.18632/aging.202627 (PMC7993734; doi:10.18632/aging.202627)
Supplement: Supplementary Figures [file aging-13-202627-s001.pdf]

SUPPLEMENTARY FIGURES

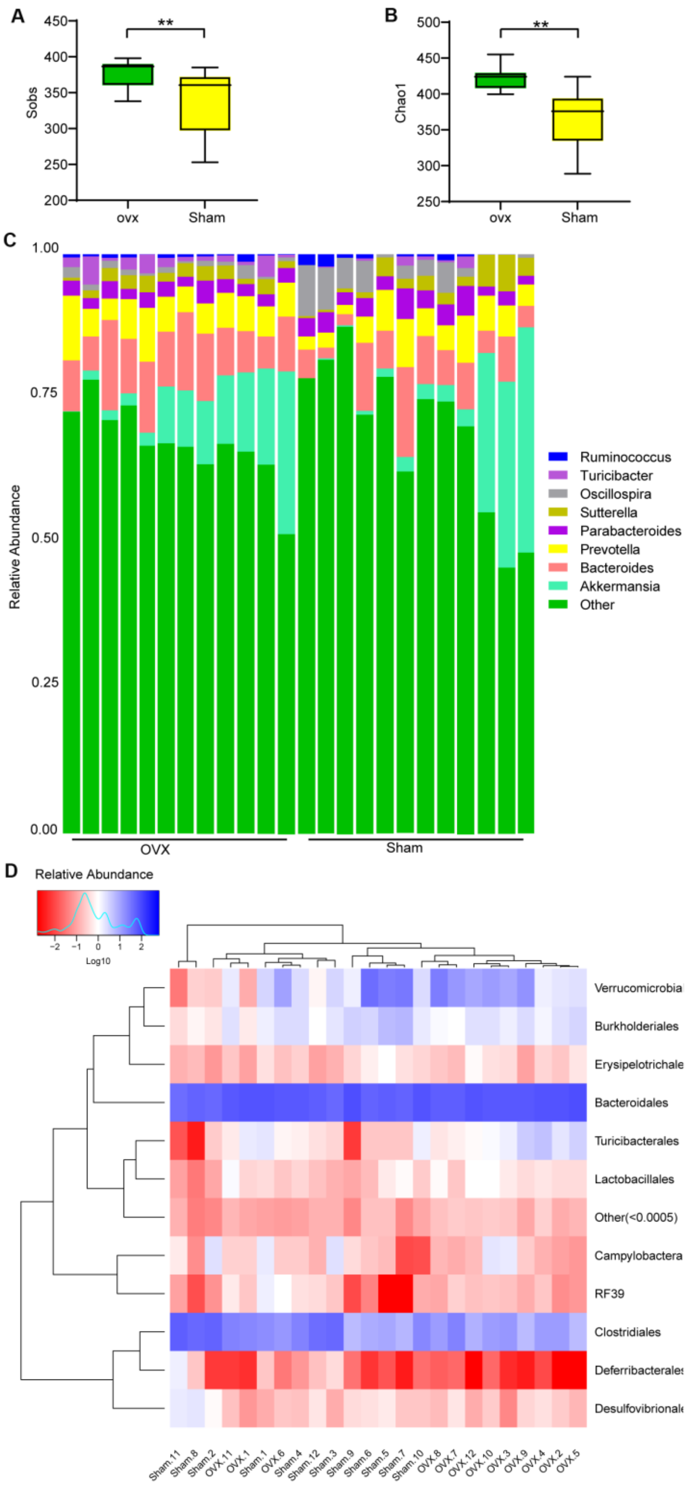

Supplementary Figure 1. Gut microbiota analysis for OVX group and Sham group. (**A**) Sobs index of α-diversity for OVX group and Sham group (t-test, $P<0.01$). (**B**) Chao 1 index of α-diversity for OVX group and Sham group (t-test, $P<0.01$). (**C**) Bar plots of fecal microbial categories at Genus level for OVX group and Sham group; (**D**) Heat map clustering based on fecal microbial categories' relative abundance at Genus level for OVX group and Sham group.

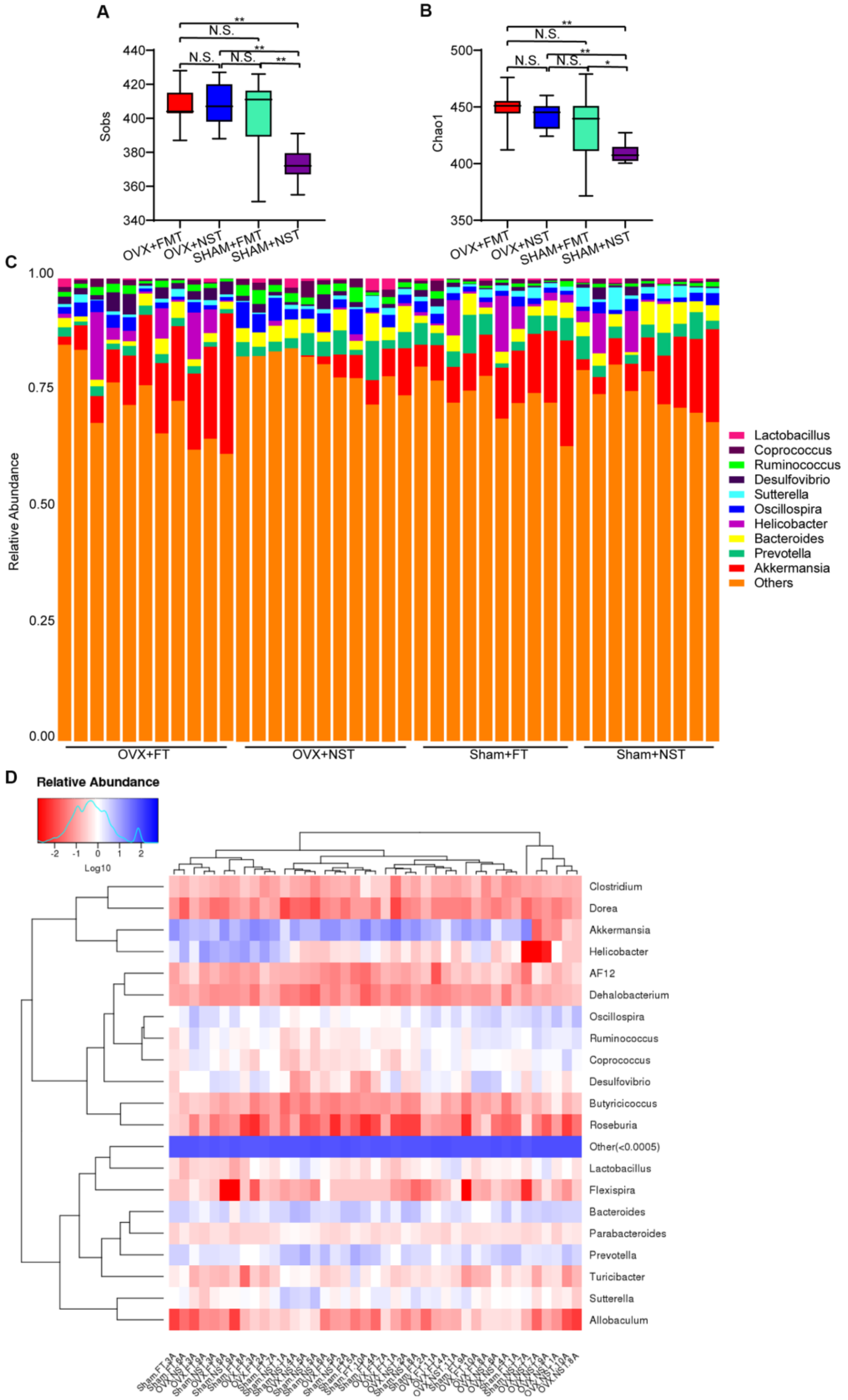

Supplementary Figure 2. Gut microbiota analysis for OVX+FMT, OVX+NST, Sham+FMT and Sham+NST group. (**A**) Sobs index of α-diversity for OVX+FMT, OVX+NST, Sham+FMT and Sham+NST groups (one-way ANOVA, $F_{3,37}$=7.54, P<0.01); (**B**) Chao 1 index of α-diversity for OVX+FMT, OVX+NST, Sham+FMT and Sham+NST groups (one-way ANOVA, $F_{3,37}$=9.28, P<0.01); (**C**) Bar plots of fecal microbial categories at Genus level for OVX+FMT, OVX+NST, Sham+FMT and Sham+NST groups; (**D**) Heat map clustering based on fecal microbial categories' relative abundance at Genus level for OVX+FMT, OVX+NST, Sham+FMT and Sham+NST groups.
